# Short-term effects of fine particulate matter on non-accidental and circulatory diseases mortality: A time series study among the elder in Changchun

**DOI:** 10.1371/journal.pone.0209793

**Published:** 2018-12-31

**Authors:** Yangming Qu, Yang Pan, Huikun Niu, Yinghua He, Meiqi Li, Lu Li, Jianwei Liu, Bo Li

**Affiliations:** 1 Key Laboratory of Zoonosis Research, Ministry of Education, School of Public Health, Jilin University, Changchun, Jilin, China; 2 Jilin Provincial Center for Disease Control and Prevention, Changchun, Jilin, China; The Ohio State University, UNITED STATES

## Abstract

**Background and objectives:**

Fine particulate matter (PM_2.5_, particulate matter with an aerodynamic diameter less than or equal to 2.5 μm) has multiple adverse effects on human health, especially on the respiratory and circulatory system. The purpose of this study was to evaluate the short-term effect of PM_2.5_ on the mortality risk of non-accidental and circulatory diseases, and to explore the potential effect modification by sex, education and death location.

**Methods:**

We collected daily mortality counts of Changchun (China) residents, daily meteorology and air pollution data, from January 1, 2014, to January 1, 2017. We focused on the elderly (≥65 years old) population who died from non-accidental causes and circulatory diseases, and stratified them by sex, education, and death location. A generalized additive Poisson regression model (GAM) was used to analyse the impact of air pollutants on mortality. We fit single pollutant models to examine PM_2.5_ effects with different lag structures of single-day (distributed lag:lag0-lag3) and multi-day (moving average lag: lag01-lag03). To test the sensitivity of the model, a multi-pollutant model was established when the PM_2.5_ effect was strongest.

**Results:**

In the single pollutant models, an increment of PM_2.5_ by 10 μg/m^3^ at lag0-3 was associated with a 0.385% (95% CI: 0.069% to 0.702%) increase in daily non-accidental mortality and a 0.442% (95% CI: 0.038% to 0.848%) increase in daily circulatory disease mortality. NO_2_ (lag1) and O_3_ (lag0, lag1, lag2, lag01,lag02, lag03) were associated with daily non-accidental death and NO_2_ (lag1, lag3, lag03) and O_3_ (lag0, lag1, lag01,lag02, lag03) were associated with daily circulatory disease mortality. In the co-pollutant models, the risk estimates for PM_2.5_ changed slightly. The excess mortality risk of non-accidental and circulatory diseases was higher for women, people with low education, and died outside hospital.

**Conclusions:**

We found that short-term exposure to PM_2.5_ increased the mortality risk of non-accidental and circulatory diseases among the elderly in Changchun. Women, people with low education and died outside hospital are more susceptible to PM_2.5_. NO_2_ and O_3_ were also associated with an increase in mortality from non-accidental and circulatory diseases and the O_3_ is a high effect.

## 1.Introduction

Ambient air pollution (AAP), which has a substantial impact on human health, has become a global public health risk[1]. Data from the World Health Organization (WHO) show that 98% of cities in the world's low- and middle-income countries experience high levels of air pollution[2]. Fine particulate matter (PM_2.5_, particulate matter with an aerodynamic diameter less than or equal to 2.5 μm) is a principal air pollutant [3]. PM_2.5_ is a mixture of various compounds that can enter major organ systems through the lungs, and it has multiple adverse effects on human health, especially on the respiratory and circulatory systems[4–8]. Numerous epidemiologic studies have reported the short-term effects of PM_2.5_[9–12]. However, short-term effects varied by exposure levels, air pollutant, population characteristics, and geographic location[13].

The WHO air quality guidelines (AQGs) set an annual mean of 10 μg/m^3^ and a 24-hour mean of 25 μg/m^3^ for PM_2.5_, and the risks of short-term and long-term mortality can be significantly reduced at below-mean concentrations[14]. Globally, the population-weighted average concentration of PM_2.5_ continues to increase, and 87% of people worldwide were exposed to PM_2.5_ levels greater than 10 μg/m^3^ in 2013[15].

In China, PM_2.5_ pollution is more severe. Only 4% of Chinese people live in areas that meet the WHO AQGs[15]. In 2013, 760,000 people in China died from exposure to PM_2.5_[16]. As the capital city of Jilin Province (China), Changchun is the largest automobile industrial city in China and the birthplace of China's automobile, film, optical, bio-pharmaceutical, and track bus industries. In 2017, the total area was 20,565 square kilometres, with a total registered population of 7.489 million and an urban population of 4.383 million. The energy source of Changchun mainly consists of coal, making it a soot polluted city. In recent years, with the increase in car ownership, there is a trend towards mixed pollution. Due to the long heating period, the air pollution in Changchun is more serious in the winter. No study on the relationship between short-term exposure to PM_2.5_ and mortality risk has been conducted in Changchun. Since the elderly are more vulnerable to air pollution[17,18], we therefore conducted a time-series study of the elderly in Changchun to evaluate the short-term effect of PM_2.5_ on non-accidental and circulatory disease mortality, and explored the potential effect modification by sex, education and death location.

## 2. Materials and methods

### 2.1. Data

#### 2.1.1. Mortality data

Daily mortality data for residents in Changchun from January 1, 2014, to January 1, 2017, was obtained from the Jilin Provincial Center for Disease Control and Prevention. The anonymous records included age, sex, date of death, and the underlying cause of death. The underlying causes of death are classified by the International Classification of Diseases (ICD-10), in which non-accidental mortality are A00-R99 and circulatory system diseases are I00~I99. To determine the daily death toll, we selected all the elderly (≥65 years old) who ‘died of non-accidental causes and those who died of circulatory diseases and stratified them by sex, education and death location.

#### 2.1.2. Air pollution and meteorology data

Daily air pollution data were obtained from ten national environmental monitoring stations in Changchun, including PM_2.5_, particulate matter with an aerodynamic diameter of equal or less than 10 μm (PM_10_), sulphur dioxide (SO_2_), nitrogen dioxide (NO_2_) and ozone (O_3_) (average concentration over 8 hours). Each indicator has at least 27 daily averages per month (at least 25 daily averages in February), and at least 324 daily averages per year. Daily meteorology data were obtained from the Changchun meteorological bureau, including average relative humidity and mean temperature. The annual data loss rate is less than 5%, and each index has at least 347 daily mean values per year. Audits of the validity, accuracy, normalization of the data, and of data quality can be used for analysis.

### 2.2. Statistical analysis

Spearman's rank correlation test was used to evaluate the correlation between air pollutants and meteorological conditions. The generalized additive Poisson regression model (GAM) was used to analyse the impact of air pollutants on the mortality of people. We applied smoothing spline functions to control the effects of confounding factors such as daily mean temperature, relative humidity, secular trend and seasonality on population mortality. The day of the week (DOW) was included in the multiple regression model as a dummy variable. The basic model was as follows:

log[E(Yi)]=βZi+s(time,ν)+s(temp,ν)+s(RH,ν)+dow+α

where:

-- *E*(*Y*_*i*_) is the expected daily death toll;
-- *s*(.) is the smoothing spline function for nonlinear variables;
-- *β* is the regression coefficient;
-- *ν* is the degree of freedom. The degree of freedom was selected according to minimum Akaike information criterion (AIC). We applied the following degrees of freedom (df): 7/year for the time trend and 3 for mean temperature and average relative humidity;
-- *dow* is the day of the week;
-- *α* is the intercept

We fit single pollutant models to examine PM_2.5_ effects with different lag structures of single-day (distributed lag: lag0-lag3) and multi-day (moving average lag: lag01-lag03). In single-day models, lag0 and lag1 for example correspond to the pollution concentration on the day and the day before. In multi-day lag models, lag03 for example corresponds to a 4-day moving average pollutant concentration of the current and previous 3 days. To test the sensitivity of the model, a multi-pollutant model was established when the PM_2.5_ effect was strongest. Considering that there may be collinearity among air pollutants, other pollutants (PM_10_, SO_2_, NO_2_, O_3_) are entered in the multi- pollutant model step-by-step. The excess risk (*ER*) was used to evaluate the increased death risk of non-accidental and circulatory diseases with each increase of 10μg/m^3^ of air pollutants (*ER* = (*RR*-1)×100%; RR, relative risk). We also stratified the association of daily mortality with PM_2.5_ by sex, education, and death location (died in hospital versus outside of hospital).

SPSS(Version 17.0; IBM Corp, Armonk, New York, USA) was used for descriptive analysis and Spearman correlation analysis, and R software (version 3.5.0; http://www.r-project.org) was used for time series analysis, with test degree α = 0.05.

## 3. Results

### 3.1. Descriptive statistics

From January 2014 to January 2017, a total of 46,780 non-accidental deaths were reported among the elderly (≥65 years old) in Changchun, of which 26,498 died of circulatory diseases. The mean daily deaths from non-accidental and circulatory diseases were 32.0 and 18.1, respectively. The daily average temperature was 7.0°C, and the daily average relative humidity was 60.1%. The daily mean concentration of PM_2.5_ was 55.9 μg/m^3^, with a Standard deviation (SD) of 49.5 μg/m^3^. For the other air pollutants, the daily mean concentrations of PM_10_, SO_2_, NO_2_ and O_3_ were 93.3 μg/m^3^, 30.7 μg/m^3^, 40.8 μg/m^3^ and 89.1 μg/m^3^, respectively (Table 1).

**Table 1:** Descriptive statistics.

Index	Mean	Standard deviation	25% quartile	Median	75% quartile	Range
**Pollution concentration**						
PM_2.5_ (μg/m^3^)	55.9	49.5	25.0	41.0	71.0	481.0
PM_10 (_μg/m^3^)	93.3	66.2	53.0	77.0	114.0	12.0
SO_2 (_μg/m^3^)	30.7	28.9	8.0	16.0	48.0	148.0
NO_2 (_μg/m^3^)	40.8	14.0	30.0	39.0	48.0	98.0
O_3 (_μg/m^3^)	89.1	38.1	58.0	82.0	113.0	212.0
**Meteorology measures**						
Mean temperature (°C)	7.0	13.8	-5.8	9.2	19.5	52.6
Average relative humidity (%)	60.1	16.8	48.0	61.0	73.0	86.0
**Daily non-accidental death counts**^**1**^						
Total	32.0	6.5	27.0	32.0	36.0	39.0
Women	15.2	4.3	12.0	15.0	18.0	26.0
Men	16.8	4.4	14.0	17.0	20.0	28.0
Junior high school and below	24.1	5.6	20.0	24.0	28.0	35.0
High school degree or above	8.0	3.1	6.0	8.0	10.0	26.0
Died in the hospital	15.8	4.3	13.0	15.0	19.0	28.0
Died outside the hospital	16.2	4.5	13.0	16.0	19.0	28.0
**Daily circulatory diseases death counts**^**2**^ **(I00-I99)**						
Total	18.1	4.7	15.0	18.0	21.0	33.0
Women	9.0	3.3	7.0	9.0	11.0	21.0
Men	9.1	3.1	7.0	9.0	11.0	21.0
Junior high school and below	14.2	4.2	11.0	14.0	17.0	29.0
High school degree or above	3.9	2.2	2.0	4.0	5.0	16.0
Died in the hospital	6.7	2.7	5.0	6.0	9.0	17.0
Died outside the hospital	11.4	3.7	9.0	11.0	14.0	24.0

^1^ people who died of all-nonaccidental causes

^2^ people who died of circulatory diseases.

### 3.2. Spearman correlation between air pollutants and weather conditions

PM_2.5_ was positively correlated with PM_10_ (*r* = 0.83), SO_2_ (*r* = 0.52) and NO_2_ (*r* = 0.72). O_3_, mean temperature and relative humidity were negatively correlated with PM_2.5_ (Table 2).

**Table 2:** Spearman correlation between air pollutants and weather conditions.

Index	PM_2.5_	PM_10_	SO_2_	NO_2_	O_3_	CO
PM_2.5_	-	-	-	-	-	-
PM_10_	0.83**	-	-	-	-	-
SO_2_	0.52**	0.37**	-	-	-	-
NO_2_	0.72**	0.57**	0.49**	-	-	-
O_3_	-0.17**	-0.03*	-0.50**	-0.11**	-	-
Mean temperature	-0.34**	-0.21**	-0.81**	-0.25**	0.70**	-
Average relative humidity	-0.02**	-0.22**	-0.05**	-0.09**	-0.23**	0.09*

*P<0.05

**P<0.01.

### 3.3. Single pollutant model

Table 3 shows the excess mortality risk of non-accidental and circulatory diseases for the elderly due to PM_2.5_ for each 10 μg/m^3^ increase on different lag days in single pollutant models. PM_2.5_ was associated with daily non-accidental mortality on lag1 and lag2 days, and the largest risk estimates were found with lag03; PM_2.5_ was associated with daily circulatory disease mortality on lag1, the largest risk estimates were also found with lag03.

**Table 3:** Association between 10 μg/m^3^ increase in PM_2.5_ and increase in deaths by lags.

Lag days	ER of daily non-accidental death count (%)	ER of daily circulatory diseases death count (%)
lag0	0.21 (-0.02, 0.44)	0.27 (-0.03, 0.57)
lag1	0.33 (0.10, 0.57)*	0.38 (0.08, 0.68)*
lag2	0.23 (0.01, 0.46)*	0.16 (-0.13, 0.45)
lag3	0.05 (-0.18, 0.28)	0.14 (-0.15, 0.43)
lag01	0.35 (0.09, 0.62)*	0.42 (0.08, 0.76)*
lag02	0.42 (0.12, 0.71)*	0.44 (0.06, 0.81)*
lag03	0.39 (0.07, 0.70)*	0.442 (0.04, 0.85)*

*P<0.05

**Fig 1:**
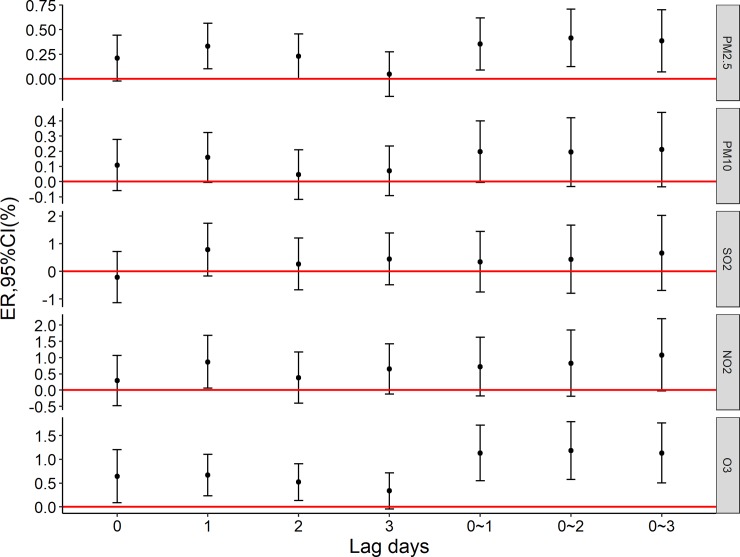
The excess mortality risk of non-accidental death per 10 μg/m^3^ increase in air pollutants.

**Fig 2:**
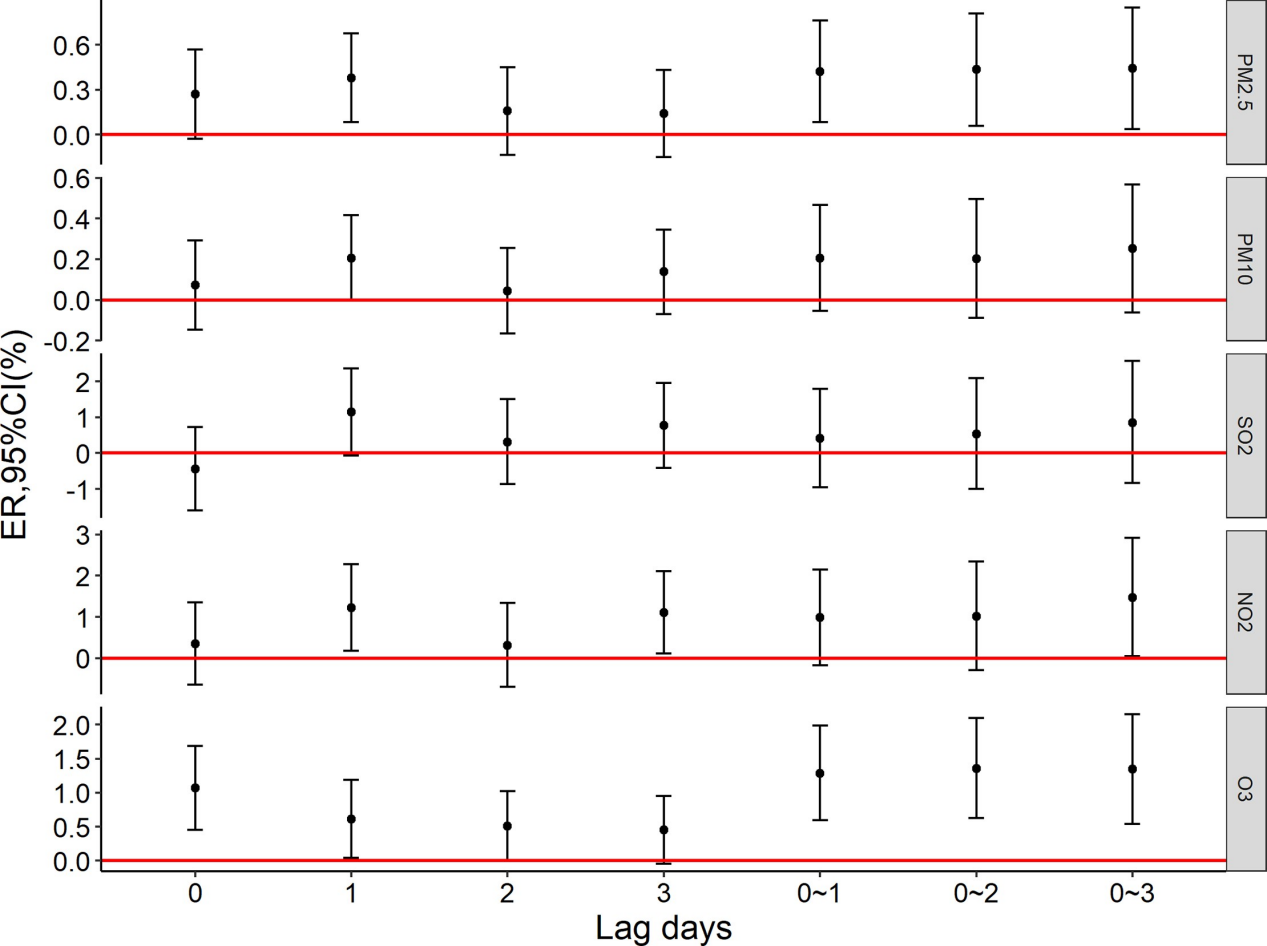
The excess mortality risk of Circulatory diseases death per 10 μg/m^3^ increase in air pollutants.

Fig 1 shows the excess mortality risk to the elderly population caused by per 10 μg/m^3^ increase in air pollutions. NO_2_ (lag1) and O_3_ (lag0, lag1, lag2, lag01,lag02, lag03) were associated with daily non-accidental death; Fig 2 shows the excess mortality risk to the elderly who die of circulatory diseases caused by per 10 μg/m^3^ increase in air pollution. NO_2_ (lag1, lag3, lag03) and O_3_ (lag0, lag1, lag01,lag02, lag03) were associated with daily circulatory disease mortality.

### 3.4. Co-pollutant model

In the single pollutant models, an increment of PM_2.5_ by 10 μg/m^3^ at lag0-3 was associated with a 0.386% (95% CI: 0.07% to 0.70%) increase in daily non-accidental mortality and a 0.44% (95% CI: 0.04% to 0.85%) increase in daily circulatory disease mortality; NO_2_ was associated with increased daily circulatory disease mortality, and O_3_ was associated with increased daily non-accidental mortality and daily circulatory disease mortality. In the co-pollutant models, the risk estimates for PM_2.5_ changed slightly (Table 4).

**Table 4:** Association between a 10 μg/m^3^ increase in PM_2.5_ (lag 03 day) and an increase in deaths using the co-pollutant model.

Pollutant model	ER of daily non-accidental death count (%)	ER of daily circulatory diseases death count (%)
**Single pollutant model**		
PM_2.5_	0.36 (0.07, 0.70)*	0.44 (0.04, 0.85)*
PM_10_	0.21 (-0.03, 0.46)	0.25 (-0.06, 0.57)
SO_2_	0.65 (-0.96, 2.02)	0.85 (-0.84, 2.57)
NO_2_	1.08 (-0.03, 2.20)	1.48 (0.05, 2.92)*
O_3_	1.13 (0.50, 1.77)*	1.34 (0.54, 2.15)*
**Co-pollutant model**		
PM_2.5_+PM_10_	0.63 (-0.03, 1.30)	0.68 (-0.18, 1.54)
PM_2.5_+SO_2_	0.39 (0.04, 0.73)*	0.43 (-0.01, 0.86)
PM_2.5_+NO_2_	0.33 (-0.08, 0.75)	0.30 (-0.23, 0.83)
PM_2.5_+O_3_	0.24 (-0.09, 0.57)	0.28 (-0.14, 0.70)
PM_2.5_+PM_10_+SO_2_	0.64 (-0.06, 1.34)	0.66 (-0.24, 1.56)
PM_2.5_+PM_10_+NO_2_	0.58 (-0.16, 1.33)	0.50 (-0.46, 1.47)
PM_2.5_+PM_10_+O_3_	0.62 (-0.05, 1.28)	0.65 (-0.20, 1.51)
PM_2.5_+SO_2_+NO_2_	0.33 (-0.08, 0.75)	0.30 (-0.23, 0.83)
PM_2.5_+SO_2_+O_3_	0.22 (-0.14, 0.57)	0.23 (-0.23, 0.68)
PM_2.5_+NO_2_+O_3_	0.18 (-0.24, 0.60)	0.11 (-0.43, 0.66)

*P<0.05

### 3.5. Subgroup analysis

The excess mortality risk of non-accidental and circulatory diseases was higher for women, people with low education and died outside hospital than it was for men, poeple with high education and died in hospital (Table 5).

**Table 5:** Association between a 10 μg/m^3^ increase in PM_2.5_ (lag 03 day) and an increase in deaths using the single pollutant model, according to sex, education and death location.

Index	ER of daily non-accidental death count (%)	ER of daily circulatory diseases death count (%)
**Sex**		
Women	0.52 (0.10, 0.94)*	0.65 (0.11, 1.19)*
Men	0.38 (-0.04, 0.80)	0.40 (-0.16, 0.96)
**Education**		
Junior high school and below	0.45 (0.09, 0.82)*	0.54 (0.08, 0.99)*
High school degree or above	0.24 (-0.36, 0.84)	0.21 (-0.61, 1.04)
**Death location**		
In the hospital	0.24 (-0.36, 0.84)	0.12 (-0.56, 0.79)
Outside the hospital	0.52 (0.10, 0.95)*	0.65 (0.15,1.15)*

## 4. Discussion

Our study analysed the short-term effects of PM_2.5_ on the elderly in Changchun. This study is the first study to examine the relationship between PM_2.5_ and population mortality in Jilin Province. We found that PM_2.5_ was associated with an increase in mortality from non-accidental and circulatory diseases. There appears to be a greater impact of PM_2.5_ on mortality from non-accidental and circulatory diseases on women, poeple with low education and died outside hospital. NO_2_ and O_3_ were also associated with an increase in mortality from non-accidental and circulatory diseases. The effect of O_3_ is higher than other pollutants.

The estimated effects in our study of PM_2.5_ on non-accidental mortality risk were lower than effects observed in a worldwide meta-analysis of a 1.04% (0.52%-1.56%) per 10 μg/m^3^ increase in PM_2.5_[19]. This discrepancy could be due to differences in population composition, geographical location and PM_2.5_ sources. However, compared with studies of Chinese populations, the estimated effects in our study are higher. For example, a recent Chinese multicity study found that the risk of non-accidental death increased by 0.22% for every 10 μg/m^3^ increase in PM_2.5_ (lag01)[20]. In another study, mortality risks for non-accidental deaths increased by 0.25% at lag days 0–1 for every 10 μg/m^3^ increase in PM_2.5_[21]. The larger effect estimates observed in our study may be due to the age of the subject. We studied the elderly and the elderly make up a high-risk group[22,23]. The short-term association of PM_2.5_ with mortality from circulatory diseases was consistent with previous epidemiological studies[17]. PM_2.5_ may reduce cardiac parasympathetic input leading to decreased heart rate variability (HRV)[24,25]. Additionally, the direct translocation of PM_2.5_ into the circulatory system can lead to an acute cardiovascular response[26–28].

For the metric of mortality from non-accidental and circulatory diseases, women, people with low education and died outside hospital were found to have increased susceptibility to PM_2.5_. Previous studies on the gender-specific health effects of air pollution are lacking, and the patterns are not conclusive. For example, Franklin et al. studied over 1.3 million deaths in 27 US communities and found that women were more susceptible than men to the mortality effects of PM_2.5_[29]. Hong et al. also found that elderly woman were more susceptible than elderly men to the mortality effects of PM_10_[30]. Similarly, a statement from the American Heart Association (AHA) concluded that particulate matter exposure may contribute to higher cardiovascular mortality in women[31]. There are some reasons that may explain the increased vulnerability of women. Inhaled particles could deposit regionally in women.[32]; women have fewer red blood cells than men, so women are more vulnerable to the toxic effects of air pollution[33]; in addition, the high reactivity of the airway to oxidants, hormonal status, smoking rates, and even a relatively low socioeconomic status are also possible causes[34]. However, the exact reasons for the gender-specific effects of air pollution on health are unclear and deserve further investigation.

Our study found that the impact on non-accidental and circulatory disease mortality from PM_2.5_ is higher for those at a lower education level than for those with a higher education level, which is consistent with previous observations on individual education level[35]. In epidemiological studies, education has been used as a surrogate indicator of socioeconomic status (SES)[36]. It is well known that SES can affect health indicators such as mortality[37]. People with a lower SES are more likely to be exposed to air pollutants and are more likely to suffer from diseases linked to air pollution that confer a greater risk of dying[38–40]. People of a lower SES may also receive inferior medical treatment and less health care, making them more susceptible to the effects of air pollution[18]. In addition, people with a lower SES may have more limited access to fish, fresh fruits and vegetables, resulting in a reduced intake of protective fatty acids and vitamins[41].

Our results showed that the impact of PM_2.5_ on mortality from non-accidental and circulatory diseases appears to be greater among people that die outside of a hospital. People outside hospital are less likely to acquire knowledge about PM_2.5_ prevention and have more exposure risks. In addition, it is more difficult for people outside hospital to receive timely treatment to alleviate a condition in the event of an outbreak.

Changchun is located in northeast China and has significant seasonal differences. Although the annual average concentration of gaseous pollutants such as SO_2_, NO_2_ and O_3_ is not high, both SO_2_ and NO_2_ maintain relatively high levels during heating periods in the winter, while O_3_ is relatively high in the summer, which exposes residents to persistent hazards from different air pollutants in different seasons. Our results showed that NO_2_ and O_3_ can increase the risk of non-accidental death in the elderly, and there was also a lag effect. Previous studies have also reached the same conclusion[42]. With co-pollutant adjustment in multi-pollutant models, the effect of PM_2.5_ on mortality changed slightly. This result suggests that our model is relatively stable.

The findings support public policy-related activities focused on the reduction of PM_2.5_ levels and the improvement of target daily PM_2.5_ standards. The estimation of the short-term mortality risk due to short-term exposure to PM_2.5_ can also help in planning appropriate medical interventions. Some limitations exist in our study: (1) Our study was based in a single city, Changchun, and generalizations need to be made in conjunction with other evidence. (2) Although it is considered reasonable to use the ambient pollutant concentration of PM_2.5_ to represent individual exposure[43], there may be errors in the PM_2.5_ exposure of different subgroups[44]. Further studies of the relationship between PM_2.5_ and mortality are needed.

## 5. Conclusions

We found that short-term exposure to PM_2.5_ increased the mortality risk of non-accidental and circulatory diseases among the elderly in Changchun. Women, people with low education and died outside hospital are more susceptible to PM_2.5_. NO_2_ and O_3_ were also associated with an increase in mortality from non-accidental and circulatory diseases and the O_3_ is a high effect.

## Supporting information

S1 Table Site coordinates. (XLSX)
